# Catalysts of violence against women students: the role of the university, aggressors, and victims

**DOI:** 10.3389/fpsyg.2024.1360192

**Published:** 2024-06-13

**Authors:** Aline dos Santos Barbosa, Marcello Romani-Dias, Tania Veludo-de-Oliveira

**Affiliations:** ^1^Schola Akadémia, São Paulo, Brazil; ^2^Fundação Getulio Vargas, Instituto de Desenvolvimento Educacional (FGV/IDE), São Paulo, Brazil; ^3^Universidade Positivo, Programa de Pós Graduação em Administração (PPGA) e Gestão Ambiental (PPGAMB), Curitiba, Parana, Brazil; ^4^Escola de Administração de Empresas da Fundação Getulio Vargas (FGV EAESP), São Paulo, Brazil

**Keywords:** university context, violence against women, women vulnerability, women strength, victims and aggressors, gender equality, quality education

## Abstract

**Purpose:**

The purpose of this study is to bring a multilevel perspective to the discussion of the antecedents of violence against women in higher education settings.

**Originality/value:**

This paper was guided by the need indicated in the literature for research on the multiple levels that constitute the context of violence against women, as this is a public health problem, a designation that indicates the urgency with which this pervasive phenomenon should be addressed. The university context is conducive to this type of research, as it includes situations that favor instances of violence. Additionally, it aligns with the United Nations Sustainable Development Goals (SDGs) of Gender Equality and Quality Educations.

**Design/methodology/approach:**

This paper follows a qualitative and interpretative approach. This choice was due to the need to know the “how” and “why” elements that are part of violence against women in the university context. As the main source of evidence for the study, we conducted 20 in-depth interviews with women (victims) and men (aggressors), all university students involved in situations of violence. The transcription of the interviews generated 346 quotations, including 41 analysis codes.

**Findings:**

After conducting the data coding, we identified that (i) the actions and omissions of the educational institution, (ii) the taste for violence, the perception of self-efficacy and the influence of the aggressors’ group of friends, and (iii) the apparent dichotomy between women’s vulnerability and women’s strength are among the main antecedents of violence against women. The article concludes with possible research questions to combat violence. Among the contribution of the discussions presented in our article, we highlight the importance of adopting a multilevel view so that we can better understand and fight against this violence, the existence of which is not restricted to the university context.

## Introduction

The fight against violence directed toward women is an urgent matter. A quarter of women worldwide aged between 15 and 24 years have been victims of gender-based violence, which takes the form of physical, sexual, psychological, and economic violence (European Institute for Gender Equality, 2019; Cepeda et al., 2022). In addition, one in three women worldwide is subjected to physical or sexual violence by partners or strangers, which corresponds to approximately 736 million women in total. The numbers are even more worrisome in low-and middle-income countries, in which more than 37% of women aged 15–49 years have suffered physical or sexual violence by a partner. Brazil, the country chosen for field research in this article, is among these countries (for more data, see the report of the World Health Organization, 2021).

The failure to prevent violence against women and to respond to women who experience violence is unscrupulous. Martin (2016) proposes the path of investigation that we used in this article. The author emphasizes that it is necessary to study multiple levels in the context of violence against women in depth, both theoretically and empirically, so that we can better act against this type of violence.

Previous studies on gender-based violence in the context of universities have had different objectives: to measure the scope of this violence in educational institutions (Benson and Thomson, 1982; Fisher et al., 1999; Bryant and Spencer, 2003; Orchowski and Gidycz, 2012; Valls et al., 2016); analyze incidence rates, types of violence, profiles of victims and perpetrators, circumstances in which violence occurs, and reactions of victims and observers (Kalof et al., 2001; Banyard et al., 2005); highlight the problem of tolerance of sexual harassment (Reilly et al., 1992; Bryant and Spencer, 2003); understand students’ perceptions of the social environments in which gender-based violence occurs (Littleton et al., 2009; Koelsch et al., 2012); explain participants’ reactions to rape prevention programs based on the Men Against Violence model (Hong, 2000; Choate, 2003); and to expand the state of the art on the mechanisms and processes that are effective (or not) for overcoming this problem outside the university context (Puigvert et al., 2019). This set of studies has contributed significantly to the creation of gender-based violence prevention programs that train students to combat violence more effectively (Coker et al., 2011).

These and other important research efforts (e.g., Colpitts, 2022; Joelsson and Bruno, 2022; Shannon, 2022) have been made to investigate specific elements that lead to violence against women in different social contexts, but to the best of our knowledge, such efforts have not yet embraced a multilevel perspective.

We found that the university context is conducive to this type of study for two main reasons. The first reason is based on the precepts of Harkins and Dixon (2010), Carey et al. (2015), Martin (2016), and Colpitts (2022), which point out the presence of multiple situations in the university context that seem to facilitate violence against female students, such as cocktail parties, parties where illicit drugs are available, the presence of fraternities and athletic teams, the hazing of freshmen, and fierce athletic competitions among universities. This context makes the statistics of violence worrying: as an example, the study by Bondestam and Lundqvist (2020) highlights that, on average, one in four female students reports having suffered sexual harassment, numbers that double, according to the authors, when we add other forms of sexual violence to the analysis. Second, the educational setting operates on multiple levels, involving the educational institution at a broader level, formal and informal groups of students at the middle level, and the victim (s) and the aggressor (s) at the individual level.

According to Bondestam and Lundqvist (2020), research on sexual harassment in higher education lacks theoretical, qualitative, and intersectional approaches and perspectives. Therefore, based on the need for a multilevel analysis of violence against women (Martin, 2016) and the relevance of the university context to this endeavor (Carey et al., 2015; Cruz, 2021; Colpitts, 2022; Shannon, 2022; Coffey et al., 2023), we ask the following questions: What are the main catalysts of violence against young university women in the university context? How do these catalysts manifest at multiple levels, and how do these levels merge to impact violence against young university women?

## Theoretical context

### In search of contributions from a multilevel perspective

Within the topic of violence against women, there are three main lines of studies on the subject in the university context: one that focused on the figure of the aggressor and the victim, one that focused on groups, and one that focused on the educational institution. For example, Abbey (2002) argues that at least half of aggression among students occurs due to alcohol consumption, which has psychological, cognitive, and motor effects on both aggressors and victims. Schrock and Schwalbe (2009) argue that the need for demonstrations of virility and masculinity reproduces gender inequalities, as men sometimes demonstrate their virility by demeaning women (McCarry, 2010). They also argue that research is needed to investigate how men encourage one another in their demonstrations of virility, relegating women to certain roles. Boyle and Walker (2016) argue that parties promoted by fraternities and athletic teams are examples of environments in which men, influenced by these subcultures, engage in violence to demonstrate their virility and later often deny that such violence has occurred. Foubert et al. (2020) contribute to this line of thinking by emphasizing that young men who participate in these groups are more likely to commit violence against women than young men who do not participate. In this scenario of violence, we must keep in mind that students who are exposed to these situations suffer physical, psychological and professional consequences. Examples such as irritation, anger, stress, discomfort, feelings of powerlessness and exclusion, are highlighted by the literature (Bondestam and Lundqvist, 2020).

Another category of studies on violence in the university context focuses on the role of institutions. For example, Holland and Cortina (2017) emphasize that, despite the increasing support that universities have provided victims of violence in recent years, victims do not seek university support for various reasons. One reason is nonexistent or inadequate communication by the university, which sometimes does not make the details of this support clear to students; another reason is victims’ perception is that it is not worthwhile to lodge complaints or ask for support because the university “would do nothing about it.” In addition, Jozkowski and Wiersma-Mosley (2017) emphasize that institutional factors, especially in contexts of class privilege, contribute to the establishment of patterns of power and control that ultimately facilitate acts of violence against women, argument also present in studies published by Phipps (2018), Colpitts (2022), and Shannon (2022) on neoliberal universities. Moylan and Javorka (2020) highlight the importance of analyzing the antecedents of violence against women at different levels in the university context, and they suggest the need for studies that investigate the culture of the institution and its context as factors that contribute to violence against women. The aforementioned works were fundamental for understanding the specific elements that precede violence against women and provided us with the opportunity to perform a multilevel analysis. In other words, although previous studies have examined important catalysts of violence against women in the university context, they have not combined the various levels of these catalysts.

We contribute to the identified gaps in the literature by performing a necessary multilevel analysis that focused on the antecedents of violence against women. Martin (2016) emphasizes that this multilevel view is fundamental for understanding the details of this type of violence; furthermore, and corroborating Harkins and Dixon (2010), Carey et al. (2015), and Colpitts (2022), we argue that the university context is conducive to this type of research, as it includes situations that favor instances of violence against women. Similarly, Jozkowski and Wiersma-Mosley (2017) argue that it is essential to analyze the factors that influence the occurrence of violence against women, as this is a public health problem, a designation that indicates the urgency with which this pervasive phenomenon should be addressed.

## Materials and methods

### The Brazilian university context

In Brazil, university students engage in activities beyond their studies, such as parties and sports games. These games are organized by the academic directories and athletic associations of the universities. Academic directories represent undergraduate students, primarily promoting social and cultural events. Athletic associations, similar to academic directories, promote university sports, organizing interclass and interuniversity championships in various modalities. During these events, students represent their universities, competing for medals and trophies. Those who do not participate as athletes can join the audience or musical groups, such as the student drums, responsible for animating the events with music. This is the context in which the higher education institution of our research is inserted, as well as its students and their situations of violence.

### The evidence

This study is the result of a broad research project on gender in organizations. Starting from a qualitative approach, the research project as a whole adopted an interpretive epistemological position because we always seek to analyze the details of certain situations, which includes focusing on the reality underlying these details and the subjective meanings that motivate human actions; therefore, there is a great emphasis on people and their feelings (Saunders et al., 2009; Burrell and Morgan, 2017).

The main data source for our article was in-depth interviews conducted with 20 students from a university that is considered elite in the Brazilian context. As selection criteria for respondents, all participants were required to be students of the educational institution and to be current or former members of a student organization. Participants are, on average, 21 years old, and are students of Business Administration, Public Administration or Economics. Most of the interviewees are in the final phase of the Undergraduate course, which contributed to a more accurate data collection due to the fact that these students have been inserted in the context of the educational institution for some years and have participated in student organizations throughout their student trajectories. The interviews were conducted between 2017 and 2018, had an average duration of 81 min, totaling 27 h, and resulted in 378 pages of transcripts, single-spaced and written in 12-point Times New Roman. We interviewed young women and young men who were studying or had studied at this institution and who were or had been members of groups of students, such as the feminist collective, the student union, the university band, and athletic teams. Specifically, we heard from nine young women from the feminist collective, the group that is responsible for collecting complaints of violence against women. We also interviewed six young women from other groups. Following the precepts of Patton (2002), we interviewed five young men who were part of student groups at the institution other than the feminist collective to confirm or refute the reports made by the young women. The young men confirmed the young women’s reports; because we found patterns in the answers that the young men provided, we concluded the interview phase of the study and began analyzing other sources to triangulate the data and increase internal validity (Lincoln and Guba, 1985; Leech and Onwuegbuzie, 2007; Creswell, 2009). Our semistructured script was organized into three categories of questions: (i) the student’s trajectory at the university; (ii) reports of violence within the university; and (iii) actions taken to respond to situations of violence. To maintain the anonymity of those involved, we use pseudonyms for all of the sources used in our article.

### Analysis

The transcription of the interviews generated 346 quotations, including 41 analysis codes. After organizing the codes, we deepened the study of the literature on violence against women to refine our categories of analysis, following the precepts of Glaser (1994) and Corbin and Strauss (2015). Then, we organized our categories of analysis in terms of catalysts of violence against women from a multilevel perspective: that of the higher education institution, with its actions and omissions; that of the aggressor and his taste for violence, perception of self-efficacy, and socioeconomic origin and the influence of friends; and that of the victims and due to the apparent dichotomy of their vulnerability and strength. Our multilevel analysis is strengthened by the fact that we also considered the groups that influence the actions of aggressors and victims and did not limit our focus to factors intrinsic to the individuals involved. In this sense, the group to which the aggressors belong (characterized as the origin of the aggressors), their group of friends at the university (characterized as influences on the aggressors), and the social group of the feminist collective (characterized as a generator of strength for victims) also constitute a substantial part of our analysis and discussion.

## Catalysts of violence against women in the university context

Young women who are university students are subjected to various types of violence (Barbosa et al., 2020). The first type is physical violence arising from the hazing of first-year students when they enter college. Jessica, who witnessed one of these events and said she was traumatized, states, “A friend of mine left the party with hypothermia because they threw a lot of beer at her. It was winter, and she was taken by the ambulance, and that only happened because she was beautiful, new to college, and the young men wanted to take advantage of her.” During other types of student events, physical violence against young women is common, as another witness points out: “I have seen young men who bit, embarrassed, scratched, and beat the young women.”

Throughout their studies at the university, these young women also suffer sexual and psychological violence. Mary showed indignation when remembering a situation she had experienced; she was with a friend at a university party, and while they were waiting in line, a young man took advantage of the chaos at the location and stuck his finger underneath the young women’s skirts. She states, “It was me and my friend, and he stuck his finger in us [...]. I screamed and then we tried to find him, but he had already gone in the midst of the crowd. I never went out in a skirt again. I was very nervous; that day was difficult. I was in shock.” In addition to such situations, freshmen hazing also provides a context for sexual and moral violence against young women. Hollander (2021) emphasizes that the vulnerability of women in certain situations makes them much more afraid of violence than men. Nguyen (2019), in turn, understands that the victim’s vulnerability can in itself create the conditions necessary for the aggressor’s action, which can intensify as the vulnerability increases.

We must always consider that violence against women generates a series of negative consequences for their lives as students. Coffey et al. (2023) point out, for example, that students who have suffered violence have persistent stress and anxiety, impaired sense of ability, and difficulties in meeting deadlines and academic expectations. It is within this difficult context of frequent violence against women that we must examine the catalysts of violence, which are center on the institution (university), the aggressors, and the victims (and social groups that interact with them).

### The university: dangerous actions and omissions

“I always have to give the young men the benefit of the doubt.” (University director, in the film *Promising Young Woman*, directed by Fennell, 2020)

“It is good for you to discuss these issues [violence against women] because they are not part of your life, but it is good for you to be concerned with the lives of the most vulnerable women. You’re from the upper class; this doesn’t happen to you.” (speech by a university administrator, reported by Margareth, an interviewee)

Real life and fiction are confused in regard to violence against women. As an illustration, the film *Promising Young Woman* (2020), winner of the 2021 Academy Award for Best Original Screenplay, portrays a situation in which the director of the university is negligent in denouncing violence by a student, a neglect that leads to impunity for the aggressors and the subsequent suicide of the victim. Unfortunately, this situation is not limited to movie screens. In real life, one of the administrators of the university where we conducted our study was also negligent in addressing violence against women, denying its existence with statements such as “they are not part of your life” and “this does not happen to you,” even in the face of ample proof of the aggression against female students and despite being part of the administration at a university that, as reported by Martin (2016), has characteristics that favor violence against women – on this point, we highlight Colpitts (2022), who warn us about the dangers of omitting violence against women. For the authors, omission is sometimes as dangerous as part of violent actions.

This university is an elite institution with a high degree of competitiveness and high academic demands; it is highly capable of investing the necessary resources to consolidate the formal and informal groups within its facilities, such as the student union and athletic teams. The existence and strength of these groups creates a situation of parties fueled by alcohol and other drugs, fierce athletic competitions, and the hazing of first-year students, a scenario that the literature indicates as one of the most conducive to violence (see classic works by Martin and Hummer, 1989; Frintner and Rubinson, 1993; Renzetti, 1996, regarding violence at these scenes). On this point, works published by Phipps and Smith (2012), Phipps (2018), and Shannon (2022), is also pertinent. As pointed out by the authors in their studies, our investigation also indicates that the university acts, to a certain extent, as a neoliberal institution that contributes to the perpetuation of male hegemony.

Competitiveness is a strong principle in this institution and is illustrated by Pietra when she describes one of the songs composed by members of the student drums: “Our mascot is the best, there is no one like it, your (competing institution) diploma is the worst one, but the best thing is when you bring my coffee; come, and I pay you minimum wage.” This competitiveness is also illustrated by the degree of difficulty of admission to the courses offered by the university. To gain admission to the university that was considered in this study, the candidate must pass a competitive entrance examination. The institution’s monthly fee is 5–10 times higher than that of other private educational institutions in the country that offer the same programs; on the one hand, this fact can fuel students’ pride in belonging to the institution, but on the other hand, it can lead them to disparage students from other institutions, especially young women.

Within this context, after entering the university, students face heavy academic demands, which can have serious consequences for them. This situation is highlighted by Andrew’s sad remembrance of a close friend who committed suicide: “One of my best friends, who was in my class, killed himself in 2015. He was feeling a lot of pressure from the university. The risk of being dismissed was great, and the college had a considerable probability of dismissing him.” Paul also highlights the effects of this heavy demand on the students’ social life; he reports, “Look, I know how to do math, I know how to run a model, but I do not know how to approach a woman.” It is within this context that university parties, most of which are promoted by student groups supported by the institution, can offer escape valves and fun for students; at the same time that they can be environments of tension and fear for young women students, culturally, they serve as a source of relief for men (Quinn, 2002; Hollander, 2021).

The institution’s omissions—situations in which it should prevent or react to violence against women but does not—also act as a catalyst for violence. Institutional neglect and tolerance of sexism are examples of this fact, as one student reports in a critical tone: “[...] we have the desire to make an anonymous denunciation channel, and the university always creates obstacles to this. Its position is always, ‘Bring the proposal for us to evaluate’. After we present the proposal, it always looks for supposed flaws and does not approve the project.” In addition to hindrances to the creation of the reporting channel, on one occasion, a student who experienced harassment and wanted to report the aggressors heard the following from managers of the institution: “Do not wear yourself out; do not try to go much further with this story. Do not let it get to the media.”

Renzetti (1996), Martin (2016), and Hurtado (2021), emphasize that it is relatively common for universities not to give due attention to these cases, partly because their administrators have multiple roles, and although they do not want violence to occur, they are negligent when they should take action. Complementarily, Gillander Gådin and Stein (2019) point out that situations of violence can be normalized at the organizational level because they are more frequent and ubiquitous in educational institutions than in other organizations. Joelsson and Bruno (2022, p.178), in turn, arrived at the following result in a study carried out in Swedish schools: “our analysis highlights a normalization of violent spatialities in young people’s everyday lives at school. Violence was normalized by framing violence as ‘unreal,’ fun and an inevitable part of growing up.” We understand that these findings are also applicable to the university context. As a result of this evidence, organizational neglect perpetuates an unequal and discouraging institutional environment for women (see Lee and McCabe, 2021, for the concept of a “chilly climate”) while also clearing the way for the actions of aggressors.

The hierarchical structure of the educational institution in our research places female students in a position of inequality. They report facing significant challenges due to this context. According to the interviewees’ reports (corroborated by the literature on the subject), there are still situations in which professors perpetuate derogatory stereotypes about women in the classroom, especially stereotypes of a sexual nature (Benson and Thomson, 1982; Marks and Nelson, 1993; Kalof et al., 2001; Bondestam and Lundqvist, 2020). This is reflected both in teaching materials, such as slides and books, as well as in inappropriate comments about students’ bodies, or even in disbelief about the performance of groups composed exclusively of women in certain academic activities, such as mathematics.

In addition, Melissa highlights the underrepresentation of female professors, evidencing structural inequality: “We have fewer female professors. This semester I did not have any, I only had 4 female professors throughout the four semesters of the course.” This lack of representation extends beyond the classroom, reaching the coordination of courses and the direction of the university, in which the female presence is minimal. Sexist comments, such as the one reported by Marcela about a law professor who justified an offensive phrase during a class, demonstrate how hostile and unequal the academic environment can be for women: “A law professor said in the context of an administrative proceeding, ‘Excuse me to feminists for what I’m going to say now, but there are women who deserve to be beaten.’ I remember the room was silent, everyone was scared.” In addition, Henrique mentions a troubling practice in which a professor shows a preference for female students by offering massages only to them during classes, while ignoring male students. “There’s a professor who, in almost every class, goes to the back of the room and gives a massage to a student, never to a male student.” These reported situations are detrimental to female students, because when harassment occurs in more established relationships between students and professors, women often lose academic self-confidence and become disillusioned with male professors. The prevalence of this type of situation has, among other things, the effect of undermining women’s possibilities in male-dominated careers (Benson and Thomson, 1982).

### The aggressors: taste for violence, self-efficacy, origin, and friendships

“O friendship all unfriendly! You strange seducer of the soul, who hungers for mischief from impulses of mirth and wantonness, who craves another’s loss without any desire for one’s own profit or revenge—so that, when they say, ‘Let us go, let us do it’, we are ashamed not to be shameless” (Augustine, 2017, 74).

“The woman is an incomplete man” (Gaarder, 2012, p. 133, referencing Aristotle).

The second group of catalysts for violence against women exhibits characteristics of the aggressors, such as a taste for sexist or violent behavior and a sense of self-efficacy that is evident in the song lyrics written by the young men. One of these songs was played by Melissa during the interview and has the following content: “I want more big breasts, I want more big asses, I want more whorehouse. I want more money in my shorts, I want more money. I want to show how it is done. I want orgy, I belong to orgy. Because I’m a whoremonger. I’m from the bacchanal. Lick my whole body, grab my dick here.” It is through the feeling of self-efficacy through aggression that the young men “want to show how it is done”; this feeling is also illustrated by a need for self-assertion, a need to present a hegemonic masculinity marked by references to superlative female erogenous zones, the drive to have physical relationships with as many women as possible (in reference to orgies), the economic power derived from the money they have, and to the male genital organ as a source of power (Barbosa et al., 2023 p.9).

This abuse among the young men can be explained by the fact that they consider this type of interaction between men and women normal. There is an element of trivialization inherent to this conduct, as if there is no problem with lyrics that place women in subordinate positions and echo Aristotle’s statement about a woman being “an incomplete man.” This reasoning is in line with the thinking of Quinn (2002), who argues that women tend to see harassment where some men see fun or normal interactions between people of different genders. Complementarily, McCarry (2010) argues that “playing violently” is one of the constituent elements of hegemonic masculinity. Based on this view, acts of violence can also be explained by ignorance on the part of young men. The trivialization of this behavior is so great that, sometimes, young men show a sense of artistic pride in the lyrics they create, without reflecting on the damage they can cause to others. This observation is evident in the report by John, a student, of a comment he made to one of the creators of a song: “Your song was so good; you sounded so cool; you made fun of the girls from other universities in a disrespectful way, but not too much.”

In addition, the aggressor’s origin appears to be an important environmental factor that catalyzes violence against women. As an illustration, the student Peter reports his context of origin when asked about situations of violence he has experienced: “I am white, a kid, straight, rich, so I think these situations practically do not happen to me, so it is difficult for me to relate.” The young man’s comment is associated with a certain normalization of situations of violence. This report does not imply that violence occurs to a greater degree among students with wealthier backgrounds, but we found that in young men, socioeconomic privileges seem to be associated with the trivialization of violence and with difficulty empathizing with victims or putting themselves in the other person’s shoes. The results of the study by Jozkowski and Wiersma-Mosley (2017), demonstrate that class privilege is a factor that can promote violence against women.

The contemporary philosopher Hannah Arendt (1906–1975), the greatest exponent of the discussion of the banality of evil, posits that there is radical evil (present in people at the top who perpetrate evil and symbolized by the hegemonic group of Nazi leaders in the context World War II) and banal evil; the latter is largely responsible for maintaining and strengthening the former and comprises a mass that closes its eyes and accepts radical evil (according to the philosopher, banal evil is present in a portion of the German population in the context of World War II concentration camps). Applying this view to our study and keeping the degree and scope of violence in their proper proportions, the young men seem to exhibit a group alienation behavior that interferes with their judgment of their own actions (see Arendt and Kroh, 1964, on the loss of capacity for judgment). From this perspective, violence can be seen as a collective privilege and not as an individual aberration (Connell, 2005).

It is within this context of collective privilege that the influence of colleagues and friends can also act as a catalyst for violence against women. In this context of influence from friends, the male students kept a diary in which they wrote drafts of new song lyrics. It contains numerous sexist, homophobic jokes and other prejudiced content. Elizabeth reported her point of view on the diary: “I am shocked when I see things that have happened, like the diary I read. They keep making fun of things, saying “oh they do not shave,” they are really childish.” In this diary, information was also written about the day when the women members of the feminist collective organized an event to talk about the importance of a less sexist and more equitable environment for female students. Elizabete says that “they wrote the report of the day of the auditorium and let it be understood that what happened at the university was the fault of the female students and they cursed them a lot, there was not much constructive in this diary.”

This reality was described in the Middle Ages by Augustine of Hippo (354–430 AD), who was later raised to the status of saint by the Catholic Church but who demonstrated violent behavior in his youth and committed robberies that were encouraged by a type of group behavior. In his book *Confessions*, he describes his behavior at the age of 16, when he stole fruits from a backyard with his friends: “I could not have done it alone. I loved it then because of the companionship of my accomplices, with whom I did it. The pleasure I got was not from the pears; it was in the crime itself, enhanced by the companionship of my fellow sinners” (Augustine, 2017, pp. 69–74). Going back to our investigation, John, one of the interviewees who had finished his university studies at the time of the interview, recalls with a certain nostalgia the complicity among friends: “I can tell you that every now and then, I still sing the songs [which were banned by the university]. When you are with people from that time, when you are drinking with friends who saw that and saw the whole process of banning that, you always end up singing.” This strength of complicity, also present in Harkins and Dixon (2010) and Martin (2016), leads the young men to meet their most primitive need to be included in the group, but at the same time, it can cause irreversible damage to someone’s life trajectory.

### The victims: the apparent duality between vulnerability and strength

“She is determined and differentiated in relation to man, while he is not in relation to her; she is the inessential in front of the essential. He is the Subject, he is the Absolute. She is the Other” (Beauvoir, 2010, 26).

“To all girls who have faced injustice and been silenced. Together, we will be heard” (Yousafzai, 2013, p. 5).

At the same time that women are subjected to the vulnerability derived from being “inessential,” as described by de Beauvoir (2010), there is also strength in being a woman; in Malala Yousafzai’s quote, this strength is characterized by their collectivity, which will ensure that “together, we will be heard.” We argue in this section that women’s vulnerability in certain contexts acts as a catalyst for violence against them. In addition, our evidence points to an apparent dichotomy, guided by the idea that a woman’s strength can also place her in circumstances that make her a victim of violence. Malala herself, a 2014 Nobel laureate, is an example of women’s strength as a catalyst for violence, as the young woman became known worldwide after she was shot in the head by the Taliban for defending the right of young women in her country to attend school. In this situation, her strength of speech and resistance made her a target of a violent extremist group, which saw her as a threat, and the terrorist who shot Malala took advantage of the fact that she was helpless, completely vulnerable, in the back seat of a school bus (Yousafzai, 2013). In this section, we will present more evidence regarding strength as a catalyst for violence; first, however, we will talk about vulnerability, which, in the interviewees’ reports, exemplifies a context of increased power of hegemonic masculinity, weakness of the rights obtained by women, and the notion of an inability to resist (on these points, see Gross et al., 2006; Schrock and Schwalbe, 2009; Jozkowski and Wiersma-Mosley, 2017).

Sarah portrays the male hegemony at the university as follows: “I can count on one hand how many female teachers I have had since the first year of the program; I think there were five, at most.” The fragility of the women’s rights is illustrated by Mary in the following statement: “I was still insecure as president (of the student union). I was not going to talk to the coordinators of the university; the young men would go, especially the general secretary and the vice president. I did not have any confidence to do things. I was [involved] in the internal management of minor things.” Patricia’s statements portray the notion of an inability to resist: “The girls had to go up on stage and do something to win the beauty contest. They said, ‘I will get you drunk; you’ll drink a lot, and you will not notice that you are up there.’ A girl took off her clothes, and the guys encouraged her. The next day they were calling her ‘bitch’.” In the reports, we noted that the young women who participated in the activities were encouraged by other people and gave in to the public’s demands for them to act the way they were expected to act, such as dancing sensually, taking off their clothes, showing intimate parts of their bodies, and kissing other young women on the lips to attain the audience’s approval and avoid retaliation.

The incidents that the young women reported confirmed can be explained with the perspectives of MacKinnon (1983) and Gross et al. (2006), who argue that women, in situations of vulnerability associated with violence committed by men, can accept this condition to avoid conflicts. When aggressors perceive that there will be no resistance on the part of the victim, they can infer that they will have an easier time successfully committing their violent acts and will not be punished for them.

In addition to the context of vulnerability in which women exist, women’s strength is also a category of analysis, which presents an apparent dichotomy. In our study, we identified two important events that illustrate women’s strength: the first is the creation of the feminist collective by an engaged group of female students, and the second surrounds the prohibition of the offensive songs composed by young men in the university band, which was an important achievement of the feminist collective and the university board and even made this prohibition official with a private institutional document in the form of communication to students, which was shown by Mariana during our interview. Both events have something in common: they seem to have fostered, for different reasons, a series of violent acts against the women involved. This overlap indicates that although women’s strength is illustrated by their conquest of rights, their spaces for speech, and their resistance, it can prompt counter resistance by aggressors (Kärreman and Alvesson, 2009; Barbosa et al., 2023). In the case of the events described above, this counter resistance was marked by strong acts of violence by male students in response to the creation of the feminist collective and the subsequent prohibition of the songs, which leads us to argue that these acts of strength on the part of women also served as catalysts for violence against them. This phenomenon is illustrated by Deborah, who was a victim of physical aggression because of her role in the feminist collective: “I became very well known as the face of feminism in college. On the one hand, it was cool because they came to me asking for help, but everyone who was against feminism came to fight with me. Once, they threw beer in my face at a college party.”

This context of violence against women intensified after the prohibition of the songs composed by young men, as Jane recalls: “I remember going to a bar, and the students did not lend me a lighter because I was a feminist, and there was a fight in the bar because of this. It was very tense.” Angela reports what she heard from the young men, “[...] ‘You oppose the words of the songs. Let us sing! You are ruining everything.’” Another student describes the violence she experienced: “There was a guy who texted me to fuck off. He said at the party that he would sing the offensive songs on the microphone using my name in them.” During the same period, women were also attacked on the way to athletic events, especially on the bus that transported the participating students. The songs were accompanied by offensive shouts, such as “screw the feminist collective,” “all feminists are fat,” and “badly fucked feminazis.” While singing these passages and harassing the members of the feminist collective, some students drew serious insults on the bus windows, as Jane reports: “They sang the song that I consider the worst of all, offending women. A boy began to say that we were Nazis (hence the use of the term “feminazi”). He drew on the bus window the symbol of the feminist collective associated with that of Nazism! I thought, ‘My God, how can this make sense in someone’s mind?’ It was very shocking!”

Our evidence regarding women’s strength as a catalyst for violence against them can be explained, in part, by the concepts of male hegemony, resistance and counter resistance present in the literature on gender. According to Hollander and Einwohner (2004), hegemony within a group—in this case, the hegemony of young men over young women—can lead to the creation of a resistance group that seeks to join forces against the dominant group and to modify a current social structure that it considers unfair. In the case of our study, the resistance group is the feminist collective, and the dominant group is represented by the young men, who exercise a hegemonic masculinity. This hegemonic masculinity in turn resists the resistance (i.e., the concept of counter resistance described in Kärreman and Alvesson, 2009).

## Conclusion

Our research offers insights into the antecedents of violence against women in the university context. Based on a multilevel analysis centered on the institution (macro level), aggressors (micro level), victims (micro level), and social groups (meso level) that interact with them, we identified, through multiple sources of evidence, that the main antecedents of violence against women include (i) the actions and omissions of the educational institution; (ii) the taste for violence, the perception of self-efficacy, the origin and the influence of friends among the aggressors; and (iii) the apparent dichotomy of vulnerability and strength in women. We argue that when a university creates an environment of high competitiveness among students and includes little female representation in the faculty and student body, while neglecting manifestations of sexism in a context of hegemonic masculinity, it creates conditions that favor and perpetuate acts of violence against female students, especially at student events outside the classroom, at gatherings, or during collective projects. Similarly, when young men take intrinsic pleasure in violence, believe that they will be successful in their aggressive actions (self-efficacy) and are strongly influenced by an origin of socio-economic privilege that places them in a position of power and by friends who think similarly, these factors may create a higher incidence of violence against women. In such situations, women are victimized because of both their vulnerability, a condition that aggressors use to their advantage, and their strength—sometimes alone, sometimes as part of a feminist collective—which can provoke strong retaliation by their aggressors. Nonetheless, feminine strength, whether individual or collective, is also fundamental for women’s protection.

The implications of these results are profound. We understand that to better combat violence against women, we need to understand its antecedents at different levels of analysis (Martin, 2016). This need was our main theoretical motivation for conducting our research. Based on our analysis and other research on this topic, we identified an urgent need for educational institutions to develop policies and actions to combat violence against women (see Bryant and Spencer, 2003; Choate, 2003; Banyard et al., 2005; Coker et al., 2011; Koelsch et al., 2012; Orchowski and Gidycz, 2012; Valls et al., 2016; Puigvert et al., 2019; Bondestam and Lundqvist, 2020). These policies should be based on intolerance of any form of violence against women, on the intervention of bystanders, and on support and solidarity with victims and their supporters. It is essential to promote more equitable spaces, with the presence not only of women in the role of professors, but also as academic managers, creating a more welcoming environment for female students. In our study, the educational institution presented difficulties in balancing the presence of female professors in the classroom and in administrative functions. This directly influences the confidence of female students in the classroom and in the environments of the institution as a whole. The students did not feel comfortable denouncing situations of violence suffered because, in addition to not having an adequate space for it, there was no presence of women to be able to dialogue. They stated that it was very embarrassing to tell a male professor or coordinator about the violence they had suffered. In particular, they stated that they were afraid of not being understood and, at times, judged as wrong or guilty for having suffered a certain situation of violence. In other words, the presence of more women, although not the only solution, is presented as one of the main means of social awareness so that situations of violence can be better combated.

To strengthen ourselves in this fight, it is necessary to implement training programs, disciplinary codes, and initiatives to prevent and raise awareness about violence against women in the university environment. Promote discussions on the subject in all spaces where situations of prejudice and discrimination may arise, such as in academic curricula and classroom debates. Take steps to deal with cases in which members of the university community are violent or harass women, including punishments for cases of false witnesses. Finally, it is necessary to create favorable environments for women, in which there is no blaming of the victims and that is permeated by solidarity, support and respect, promoting the encouragement of victims and others to report cases of violence and seek assistance, in addition to promoting the intervention of bystander.

Continued research at these and other levels of analysis is necessary, as are studies that address the important relationships among antecedents of violence, to make the fight against violence more effective. The categories of analysis that emerged from our sources of evidence may directly contribute to future research, and we propose the following research questions to combat violence: How can women’s strengths generate protective actions and prevent omissions by the institutions to which they belong? How can these same female strengths decrease the perception of self-efficacy and the taste for violence among aggressors? How can the search for more inclusive and representative environments for women at institutions reduce the effects of compromising friendships and the notion of impunity regarding the actions of possible aggressors? How does this type of environment contribute to making women less vulnerable? These issues may guide research in the university environment or in other contexts that are also prone to violence. We also consider relevant studies that examine the relationship of women’s strength and vulnerability as antecedents of violence against them. In this scenario, research addressing the phenomenon of counter resistance, which is marked by strong retaliation by men who wish to maintain their male hegemony in the face of women’s increasing strength, is opportune. As a last topic, more empirical research is needed on educating young people against gender-based violence. These studies can be based on the theoretical article by Mclean (2023), for whom the fight against gender violence involves developing self-reflection and empathy in young people.

## Data availability statement

The datasets presented in this article are not readily available because the data cannot be made available due to confidentiality agreements with the participants and the educational institution. Requests to access the datasets should be directed to aline8barbosa@gmail.com.

## Ethics statement

Ethical approval was not required for the studies involving humans because the present study, conducted in the field of administration, did not undergo ethical review by an ethics committee, as it falls outside the scope of research that typically requires such approval. In accordance with established ethical standards, certain areas of study, including administrative research, may not be subject to mandatory ethical committee approval. However, the research adheres to ethical principles, ensuring confidentiality, respect for participants, and responsible data handling throughout the study. If you have any concerns or questions regarding the ethical aspects of this research, please feel free to contact the research team. The studies were conducted in accordance with the local legislation and institutional requirements. The participants provided their written informed consent to participate in this study. Written informed consent was obtained from the individual(s) for the publication of any potentially identifiable images or data included in this article.

## Author contributions

AB: Conceptualization, Data curation, Formal analysis, Investigation, Methodology, Software, Writing – original draft, Writing – review & editing. MR-D: Conceptualization, Data curation, Investigation, Methodology, Writing – original draft, Writing – review & editing. TV-d-O: Conceptualization, Formal analysis, Resources, Supervision, Writing – review & editing.
